# Beneficial effects of a novel RAGE inhibitor on early diabetic retinopathy and tactile allodynia

**Published:** 2011-12-06

**Authors:** Guangyuan Li, Jie Tang, Yunpeng Du, Chieh Allen Lee, Timothy S. Kern

**Affiliations:** 1 Case Western Reserve University, Cleveland, OH; 2 The Second Hospital of Jilin University, Changchun, Jilin, China; 3 Cleveland VAMC Research Service 151, Cleveland, OH

## Abstract

**Purpose:**

The receptor for advanced glycation end products (RAGE) has been implicated in the pathogenesis of numerous complications of diabetes. We assessed the effect of a novel RAGE fusion protein inhibitor on retinal histopathology and nerve function, and on retinal inflammation and oxidative stress.

**Methods:**

C57BL/6J mice were made diabetic with streptozotocin, and some were given a RAGE fusion protein (10, 100, or 300 µg per mouse 3 times per week). Mice were sacrificed at 2 months and 10 months into the study to assess retinal vascular histopathology, accumulation of albumin in the neural retina, cell loss in the ganglion cell layer, and biochemical and physiologic abnormalities in the retina. Tactile allodynia (light touch) was measured on a paw of each animal at 2 months.

**Results:**

Leukostasis, expression of the intercellular adhesion molecule-1 (ICAM-1), accumulation of albumin in the neural retina, and nitration of retinal proteins were significantly increased in the retinas of mice diabetic for 2 months. The number of degenerate retinal capillaries was significantly increased in mice diabetic for 10 months, compared to the nondiabetic controls. Diabetes also enhanced sensitivity of peripheral nerves to tactile allodynia. All three doses of the RAGE fusion protein inhibited capillary degeneration, accumulation of albumin in the neural retina, nitration of retinal proteins, and tactile allodynia, demonstrating that biologically meaningful levels of the drug reached the retina. RAGE inhibition did tend to inhibit diabetes-induced retinal leukostasis and ICAM-1 expression (previously postulated to be important in the pathogenesis of retinopathy), but these effects were not statistically significant for the use of the lower doses of the drug that normalized the vascular histopathology.

**Conclusions:**

Inhibition of RAGE blocked the development of important lesions of diabetic retinopathy, but these beneficial effects seemed not to be mediated via leukostasis. RAGE inhibition also blocked the development of sensory allodynia in diabetes. RAGE is an important therapeutic target to inhibit the development of vascular and neural complications of diabetes.

## Introduction

Retinopathy is a common complication of diabetes, and is the principal cause of blindness in the adult population. Biochemical abnormalities postulated to contribute to the development of this retinopathy have been numerous [1-5], including signaling via advanced glycation endproducts (AGEs) and the receptor for advanced glycation end products (RAGE). Increased formation of AGEs is one of the best-recognized biochemical abnormalities of diabetes. RAGE is a multiligand receptor that mediates many or all of the sequelae of AGEs interacting with the cell surface, but it also binds other ligands, including S100/calgranulins, amphoterin/High mobility group box 1 (HMGB1), and amyloid fibrils. AGEs interact with RAGE to induce proinflammatory, pro-adhesive, and growth-stimulating signals, and these changes have been associated with or causally linked to abnormalities in several cell types and tissues [2,6-8].

The AGE:RAGE axis is also active in the retina [2,9,10]. Soluble RAGE (sRAGE), a secreted isoform that acts as a competitive inhibitor of AGE-mediated alterations in cells, has been shown to inhibit diabetes-induced changes in retinal histology and in electroretinograms produced with an experimental model of diabetic retinopathy [9]. Since a growing body of work implicates inflammation and nitric oxide in the development of the early stages of diabetic retinopathy [4,5,11-15], we evaluated the effects of a new fusion protein inhibitor of RAGE signaling (RAGE-Ig fusion protein) on the development of diabetes-induced alterations in retinal physiology, inflammation and histopathology in C57Bl/6J mice. To determine whether or not the effects of the drug were limited to retinas in diabetes cases, we also assessed the effects of the drug on a parameter of diabetes-induced sensory neuropathy (sensitivity to light touch, i.e., tactile allodynia) in peripheral nerves.

## Methods

### Experimental animals

Male C57Bl/6J mice were randomly assigned to become diabetic or remain untreated in the nondiabetic group. Diabetes was induced by five sequential daily intraperitoneal injections of a freshly prepared solution of streptozotocin in citrate buffer (pH 4.5) at 60 mg/kg of bodyweight. After hyperglycemia was verified at least three times during the second week after streptozotocin, diabetic mice randomly were assigned to be untreated diabetic controls or to be administered the RAGE-Ig fusion protein intraperitoneally at three different concentrations (10, 100, and 300 µg per mouse) three times per week. We observed no adverse effects of any dose of the RAGE-Ig fusion protein on the bodyweight gain or overall health of the diabetic mice. Insulin was given as needed to prevent weight loss, but without preventing hyperglycemia or glucosuria (0.0–0.2 units of Neutral Protamine Hagedorn insulin subcutaneously, zero to three times per week). Glycohemoglobin was measured using the Bio-Rad Total Glycated Hemoglobin Assay (Bio-Rad, Hercules, CA) every 2–3 months and just before the animals were killed. Food consumption and bodyweight were measured weekly. The animals were studied either for a 10-month duration of diabetes to investigate the effects of the therapy on the retinal histopathology, or for a 2-month duration to investigate the therapy’s effects on molecular and physiologic changes. The treatment of all animals used in this study conformed to the ARVO Resolution on the Treatment of Animals in Research.

The RAGE fusion protein (provided by L. Brown, Galactica Pharmaceuticals, Villanova, PA) consists of a RAGE ligand-binding element, a heavy-chain immunoglobulin of a G4 isotype constant domain, and a linker connecting the ligand-binding element with the constant domain. The RAGE-Fc (Fragment, crystallizable region of the antibody) binds to all of the known ligands of RAGE, and acts as a competitive, negative regulator of RAGE signaling by competing with membrane-bound receptors for binding ligands. As demonstrated previously [16], the binding of the soluble RAGE to its ligands inhibits the binding of those ligands to membrane-based RAGEs. The available evidence indicates a strong affinity of this RAGE fusion protein for HMGB1 (not shown), and we speculate that most of the effects seen with this inhibitor are mediated through the binding of HMGB1. For the present study, a murine version of human RAGE was employed in which a G2a isotype constant domain (biologically equivalent to the G4 isotype in the hRAGE) was used. The molecule was created in Chinese hamster ovary cells cells using the GPEx expression system (Gala Biotech, Middleton, WI). The molecule has a molecular weight of approximately 140 kDa.

### Diabetes-induced retinal histopathology

After 10 months of diabetes, the mouse eyes were fixed in formalin, and one retina from each animal was isolated, washed in running water overnight, and digested for 2 h in Difco crude trypsin solution (Cat no. 15090–046; Invitrogen Corp., Carlsbad, CA), as we have reported previously [17-20]. When totally cleaned of neural cells, the isolated vasculature was laid out on a glass microscope slide, dried overnight, stained with hematoxylin and periodic acid (Schiff base; Sigma-Aldrich, St. Louis, MO), dehydrated, and coverslipped. Degenerate (acellular) capillaries were quantitated in six to seven field areas corresponding to the midretina (200× magnification) in a masked manner. Acellular capillaries were identified as capillary-sized vessel tubes having no nuclei anywhere along their length, and were reported per square millimeter of retinal area. Pericyte “ghosts” were estimated from the prevalence of protruding “bumps” in the capillary basement membranes from which pericytes had disappeared. At least 1,000 capillary cells (endothelial cells and pericytes) in five field areas in the mid-retina were examined (at 400× magnification) in a masked manner. Ghosts on any already acellular vessels were excluded.

To study the effects of diabetes on retinal neurodegeneration, the cells in the ganglion cell layer were counted. The formalin-fixed eyes were embedded in paraffin, sectioned sagittally through the retina including through the optic nerve, and stained with hematoxylin-eosin. The number of cells in the ganglion cell layer was counted in two areas (the midretina and the posterior retina adjacent to the optic nerve) on both sides of the optic nerve. Comparable areas from both sides of the optic nerve were averaged and expressed per unit length.

### Adhesion of white blood cells to retinal blood vessels

After 2 months of diabetes, blood was removed from the vasculature of anesthetized animals by complete perfusion with phosphate-buffered saline (0.01 M phosphate buffer, 0.0027 M potassium chloride and 0.137 M sodium chloride, pH 7.4; Sigma-Aldrich, St. Louis, MO) via a heart catheter. Animals were then perfused with fluorescein-coupled concanavalin A lectin (20 μg/ml in PBS; Vector Laboratories, Burlingame, CA), as described previously by us and others [19,21-23]. Flatmounted retinas were imaged via fluorescence microscopy, and the number of leukocytes adherent to the vascular wall was counted.

### Leakage of albumin into neural retina

The amount of leakage of albumin into the neural retina was used to estimate vascular permeability. After 2 months of diabetes, the eyes were cryosectioned (section thickness: 10 µm), fixed in methanol for 10 min, and washed (4× in PBS). Each section was incubated in sheep antimouse serum albumin (AB8940; 1:2,000 dilution; Abcam, Cambridge, MA) for 2 h. After washing, sections were incubated in fluorescein isothiocyanate-labeled secondary antibody for 90 min (AB 6743; 1:1,000 dilution; Abcam). Under fluorescence microscopy, the average amount of fluorescence was determined in each of four retinal layers (the inner plexiform layer, inner nuclear layer, outer plexiform layer, and outer nuclear layer). Measurements were made in three replicate areas in each retinal layer, both at midretina and beside the optic nerve. The amount of fluorescence at each site was the average of ten replicated measurements taken using software (NIS Elements 126 BR 3.00; Nikon Instruments, Inc., Melville, NY).

### Nitration of retinal proteins

After 2 months of diabetes, retinas were isolated and homogenized. Dot blots were made, blotting 50 µg protein homogenate from each animal onto nitrocellulose membranes. The membranes were blocked with milk (5%), washed, and immunostained using antinitrotyrosine (#05–233; 1:500 dilution; Upstate Biotechnology, Lake Placid, NY) for 2 h, and then stained with secondary antibody (goat anti-mouse IgG-HRP conjugate; 1:1,000 dilution; 1 h; Bio-Rad). After extensive washing, immunostaining detected by the antibody was visualized by enhanced chemiluminescence (Santa Cruz Biotechnology, Santa Cruz, CA). The immunostain-dependent chemiluminescence was recorded on film, and the density of the immunostained dots was quantitated. Results were expressed as a percent of values detected in the nondiabetic controls.

### Expression of retinal intercellular adhesion molecule-1 and cyclooxygenase-2

Retinas were isolated, sonicated, and centrifuged, and the supernatant was used for western blots. Samples (50 µg) were fractionated by sodium dodecyl sulfate PAGE (SDS–PAGE) and electroblotted onto nitrocellulose membranes. The membranes were blocked in Tris-buffered saline containing 0.02% Tween-20 (Sigma-Aldrich, St. Louis, MO), and 5% nonfat milk. Antibodies for ICAM-1 and cyclooxygenase (COX)-2 (1:200 dilution; Santa Cruz Biotechnology) were applied, followed by secondary antibody, for 1 h. After washing, the results were visualized with enhanced chemiluminescence.

### Tactile allodynia

A standardized testing regime was performed to measure tactile allodynia, as reported previously [24-26]. Animals were studied after 8 months of diabetes. A series of Von Frey filaments (Stoelting, Wood Dale, IL), starting with one that possessed a buckling weight of 2.0 g, were applied in sequence to the plantar surface of the right hindpaw with a pressure that caused the filament to buckle. The lifting of the paw was recorded as a positive response, and the next lightest filament was chosen for the next measurement. The absence of a response after 5 s prompted use of the next filament of greater weight. This process was continued until four measurements had been made after an initial change in behavior, or until five consecutive negative scores (a score of 15 g) or four positive scores (a score of 0.25 g) had resulted. The resulting sequence of positive and negative scores was used to interpolate the 50% response threshold, as described previously. All measurements were performed by an investigator who was unaware of the treatment group of individual animals.

### Statistical analysis

Data were expressed as mean±standard deviation (SD). Statistical analysis was performed using an ANOVA (ANOVA), followed by Fischer’s test. A value of p<0.05 was considered statistically significant.

## Results

Glycemia was elevated to a similar level in all diabetic groups. The average glycated hemoglobin levels over the entire duration of the 10-month experiment were 3.2±0.2%, 12.5±0.4%, 12.1±0.8%, 12.6±0.9%, and 12.6±0.8 for the nondiabetic, diabetic control, and diabetic groups (treated, respectively, with 10, 100, and 300 µg mRAGE-Fc). Final bodyweights in these same groups were 44±6.7 g, 27±2 g, 27±3 g, 26±3 g, and 27±2 g, respectively. Data from the two-month experiment were similar (but not shown). Administration of the mRAGE-Fc had no effect on the glycemia or health of the animals at any of the doses administered.

### Inhibition of diabetes-induced retinal histopathology by inhibitor of receptor for advanced glycation endproducts

**Figure 1:**
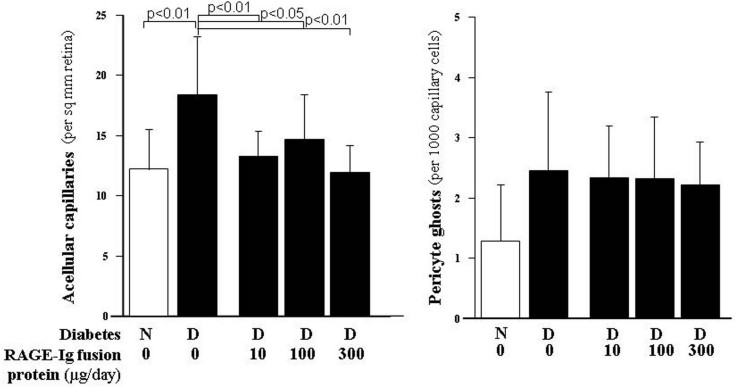
Administration of the RAGE-immunoglobulin fusion protein for 10 months inhibited the diabetes-induced increase in degenerate, acellular capillaries. The inhibitor was administered at three doses, 10, 100, 300 µg/day, for the entire duration of the study. Pericyte loss (as indicated by ghosts where the pericytes used to be) also tended to be increased by diabetes, but the inhibitor did not significantly reduce their formation. All groups contained at least ten animals.

Long-term diabetes resulted in a significant increase in the number of degenerate, acellular capillaries in the retinas of the control animals. Figure 1 shows that all three doses of the RAGE-Ig fusion protein significantly inhibited the diabetes-induced degeneration of retinal capillaries. Diabetes tended to increase pericyte degeneration (pericyte ghosts), but the results from these animals did not achieve statistical significance. Neither did diabetes induce a significant decrease in the number of cells in the retinal ganglion cell layer (i.e., neurodegeneration); therefore, we could not assess any effects of the RAGE inhibitor on either of these parameters (not shown).

In an effort to investigate the mechanism by which the therapy mediated this beneficial effect on the degeneration of retinal capillaries in diabetes cases, we measured several parameters related especially to inflammation and nitrative (oxidative) stress. These were physiologic and molecular abnormalities that have been found in other studies to be associated with (and possibly causally related to) the development of the early stages of diabetic retinopathy.

### Intercellular adhesion molecule-1 and leukostasis

**Figure 2:**
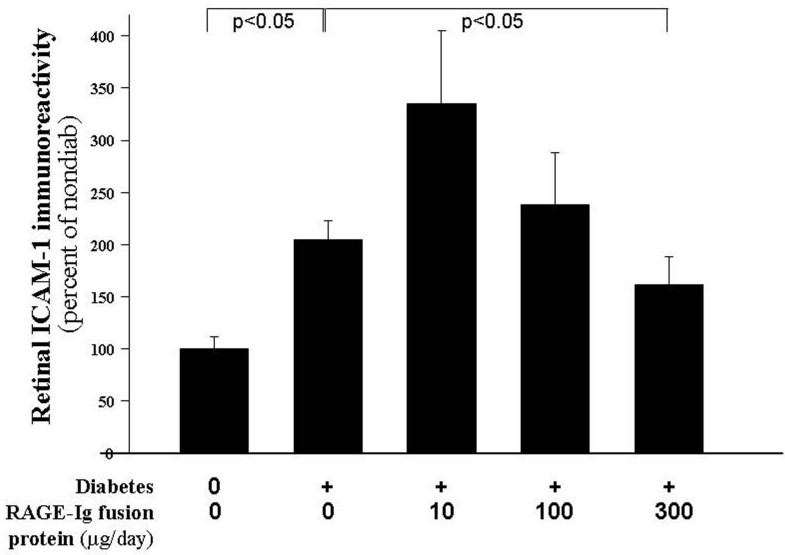
Only the highest dose of the receptor for advanced glycation endproducts (RAGE) inhibitor significantly inhibited the diabetes-induced increase in retinal intercellular adhesion molecule-1 (ICAM-1) expression. Diabetics were treated with 0, 10, 100, or 300 μg/day of the RAGE-Immunoglobulin fusion protein. Only the highest dose of inhibitor significantly inhibited retinal ICAM-1 expression below that seen in control diabetics. The duration of diabetes and drug administration was 2 months. n=6–10 in all groups.

**Figure 3:**
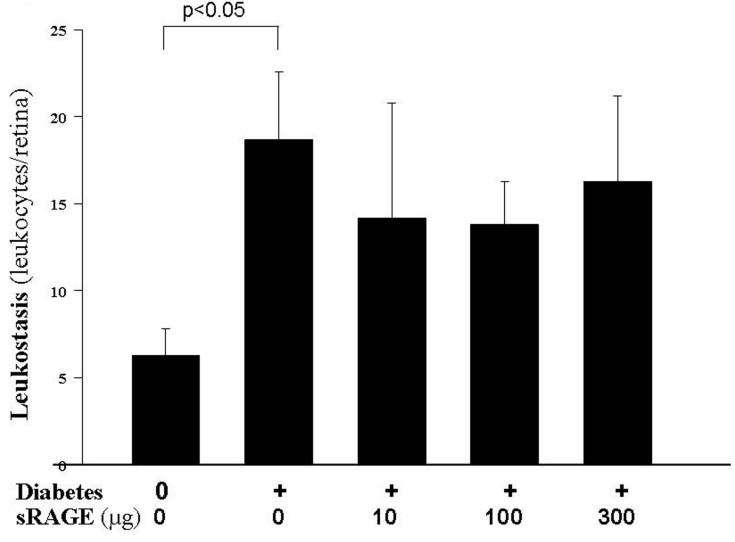
RAGE inhibition does not inhibit leukostasis in the retinal vasculature. Diabetes (after months’ duration) significantly increased the adherence of leukocytes to the retinal vasculature, and the RAGE-Ig fusion protein tended to inhibit this increase, but the extent of inhibition was not statistically significant at any dose. n=6–10 in all groups.

Since ICAM-1 expression on endothelial cells plays a critical role in inflammation through the adhesion of white blood cells to the vessel wall (leukostasis), we measured the effect of diabetes and the therapy on the expression of ICAM-1 in retinas and on leukostasis. Figure 2 and Figure 3 show that diabetes of two months’ duration resulted in a significant increase in the expression of retinal ICAM-1 and in the number of leukocytes adherent to the retinal blood vessels (p<0.05). Administration of the RAGE-Ig fusion protein resulted in a dose-dependent effect on the expression of ICAM. The highest dose significantly inhibited this expression, although the lowest dose actually increased the expression of the adhesion molecule above that observed in the diabetic controls. Leukostasis tended to be inhibited in these animals treated with the RAGE-Ig fusion protein, but the inhibition did not achieve statistical significance.

### Accumulation of albumin in neural retinas

**Figure 4:**
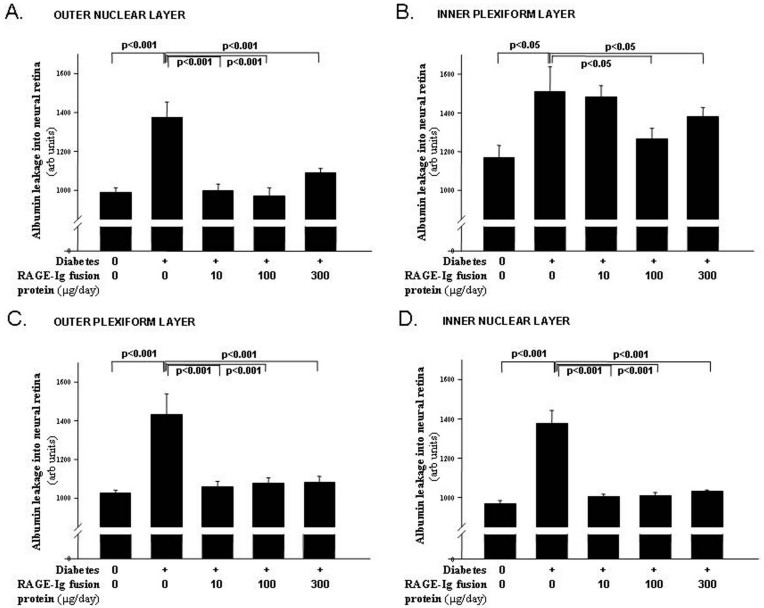
RAGE-Ig fusion protein inhibited albumin accumulation in the neural retina. Diabetes resulted in a significant increase in the levels of immunoreactive albumin in the nonvascular retina (i.e., between vessels) in the **A**: outer nuclear layer, **B**: outer plexiform layer, **C**: inner nuclear layer, and **D**: inner plexiform layer. All three treatment doses doses normalized albumin leakage in all layers except in the inner plexiform layer, where only the two highest doses of the RAGE-Ig fusion protein significantly inhibited the leakage of albumin. n=6–10 in all groups.

Diabetes after 2 months resulted in a significant increase in the levels of immunoreactive albumin in the nonvascular retina (i.e., between vessels) in each of the four retinal layers studied (Figure 4A-D). Accumulation of the blood protein, albumin, in the neural retina has been viewed as a marker of increased vascular permeability. In the outer nuclear layer (Figure 4A), outer plexiform layer (Figure 4B), and inner nuclear layer (Figure 4C), all three doses of the RAGE-Ig fusion protein significantly inhibited the accumulation of albumin in the neural retina. In the inner plexiform layer (Figure 4D), the 100 µg and 300 µg doses of the RAGE-Ig fusion protein significantly (but only partially) inhibited albumin leakage, while the 10 µg dose had no effect.

### Nitration of retinal proteins and cyclooxygenase-2 expression

**Figure 5:**
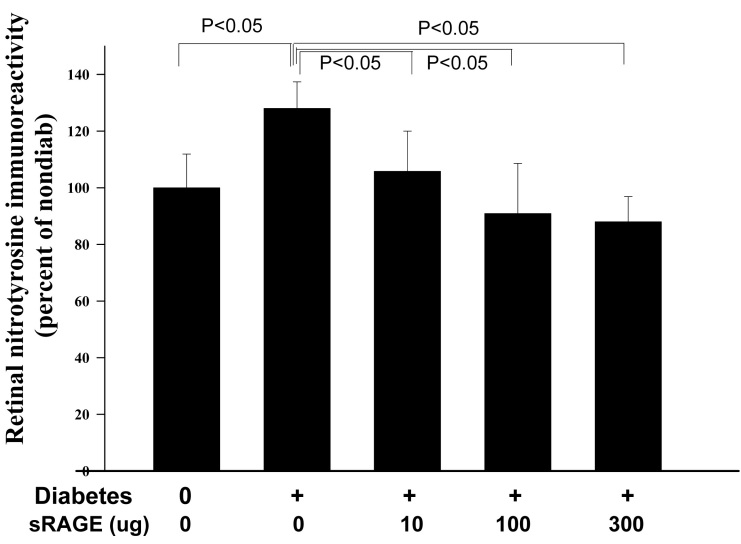
RAGE-Ig fusion protein inhibited nitration of retinal proteins (assessed as nitrotyrosine modification of retinal proteins). All three doses of the inhibitor significantly inhibited the nitration of retinal proteins compared to that in diabetic controls. The duration of diabetes was 2 months. n=6–10 in all groups.

The nitration of proteins is regarded as a parameter of both oxidative and nitrative stress. Figure 5 shows that retinas from diabetic mice exhibited a significant increase in the nitration of retinal proteins, compared to the nitration levels detected in retinas from the nondiabetic animals (p<0.05). The RAGE inhibitor significantly inhibited, in a dose-dependent manner, this post-translational modification. Expression of the protein band corresponding to COX-2 did not increase in diabetic animals and did not change in animals getting the therapy (not shown).

### Tactile allodynia

**Figure 6:**
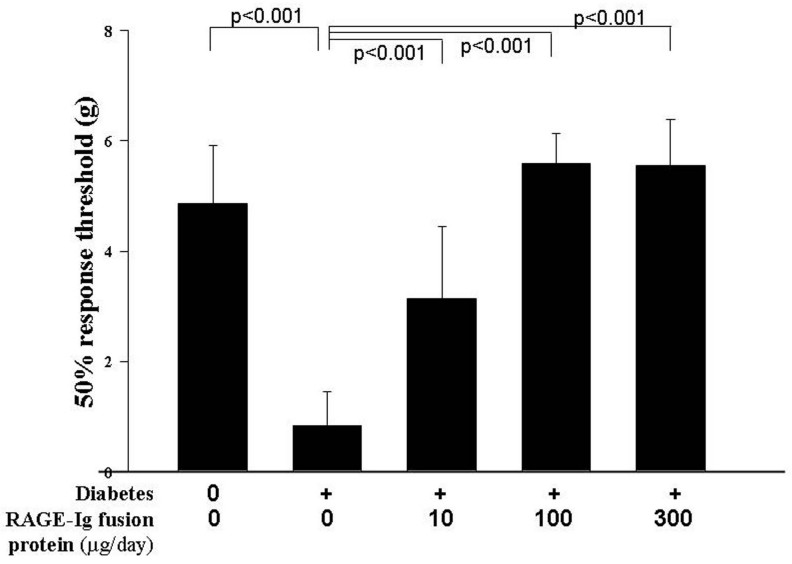
RAGE-Ig fusion protein inhibited the diabetes-induced hypersensitivity to tactile allodynia (light touch). Diabetic controls moved their legs at a lower pressure, indicating that they had increased sensitivity to touch. The inhibitor was administered at three doses of 10, 100, and 300 µg/day, and the duration of diabetes and drug administration was 2 months. n=6–10 in all groups.

The beneficial effects of this RAGE inhibitor in diabetes were not limited to the retina. Figure 6 shows that diabetes enhanced the sensitivity of peripheral nerves to tactile allodynia in the diabetic control mice, and administration of the two highest doses of the RAGE inhibitor significantly inhibited the diabetes-induced hypersensitivity of this sensory function. The lowest dose of drug (10 µg) had a partial yet still significant effect.

## Discussion

RAGE has been postulated to contribute to diabetic complications by binding a variety of molecules, including AGEs, HMGB1, S100, and integrins [6,8,27-29]. Such interactions lead to inflammatory processes, including the upregulation of adhesion molecules, generation of cytokines and reactive oxygen species, and upregulation of monocyte chemoattractant protein 1, at least in part through the activation of NF-ĸB [7,8,30-37]. The mechanisms by which RAGE inhibitors have been postulated to work include by being a scavenger for soluble AGEs, by acting as a competitive antagonist for membrane-bound RAGEs and for the full-length biologically active RAGEs, and by binding HMGB1 to prevent its interaction with membrane RAGEs [16].

The RAGE inhibitor fusion protein was able to inhibit important histopathology in the retinas of diabetic mice—a finding consistent with a prior study in which early retinopathy was inhibited using sRAGEs as competitive inhibitors of RAGE binding [9]. We have extended the findings of that study by also demonstrating the successful use of a fusion protein to inhibit the histopathology, and by demonstrating that the drug’s effects on histopathology seem not to be explained by its effects on several molecular changes previously postulated to be important in the pathogenesis of retinopathy.

An important feature of our study was that we tested the therapy with three dosage levels. All three doses of the therapy tested significantly inhibited the diabetes-induced degeneration of the retinal vasculature. Likewise, all three doses of the drug also seemed to inhibit any diabetes-induced increase in albumin accumulation in the neural retina. Surprisingly, some abnormalities that have previously been postulated to contribute to the development of retinal vascular injury and retinopathy were not similarly inhibited by all doses of our RAGE inhibitor.

Prior studies have demonstrated that RAGE serves as an adhesion receptor that interacts with integrins and facilitates the recruitment of proinflammatory leukocytes at the sites of inflammation, further enhancing the inflammatory state [27,38-40]. Moreover, diabetes induces RAGE on retinal Müller cells [9,41], endothelial cells [42], and leukocytes [43]. Given these observations, an unexpected finding in our studies was that doses of mRAGE-Fc that had little or no apparent effect on several parameters of inflammation (e.g., leukostasis, ICAM expression), nevertheless significantly inhibited diabetes-induced capillary degeneration. The lack of any effect of the present RAGE inhibitor on leukostasis seems inconsistent with the earlier report that the sRAGE did inhibit the increase in leukostasis, in vitro, in diabetes cases [38,42]. Nevertheless, evidence that we generated previously [20] suggests that leukostasis, per se, is unlikely to be causal in the development of early diabetic retinopathy in rodents.

Although the effects of RAGE inhibition on ICAM-1 and leukostasis did not always parallel its effects on capillary degeneration, the effects of the drug on nitrative stress and leakage of albumin were closely associated with capillary degeneration. All three doses of the RAGE inhibitor blocked protein nitration, leakage of albumin into the neural retina, and capillary degeneration. Increased vascular permeability in the retinas from diabetic animals has been linked to the presence of nitric oxide [13], so it might be that the inhibition of albumin leakage detected by us using the RAGE inhibitor was secondary to the effects from nitric oxide generation. Previous studies by us and others have demonstrated that the diabetes-induced degeneration of retinal capillaries in animals is essentially prevented in diabetic mice deficient in the inducible isoform of nitric oxide synthase, or is prevented if wild-type diabetics are given aminoguanidine, an inhibitor of the nitric oxide synthase [17,18,44-46].

AGE–RAGE interactions have been shown to induce oxidative stress, to upregulate NF-ĸB and related proinflammatory genes in peripheral nerves, and to exaggerate neurologic dysfunction [47-49]. Moreover, diabetic mice (like human patients) develop altered nerve function [50], and diabetic mice lacking RAGE have attenuated features of neuropathy and limited activation of potentially detrimental signaling pathways [51]. Therefore, we investigated the effect of the RAGE inhibitor on the dysfunction of peripheral nerves in diabetes cases. Since an appreciable number of diabetic patients develop hypersensitivity to touch or heat (which is not to be confused with the reduced peripheral sensitivity that also develops in diabetes), we assessed effects of the drug on sensitivity to tactile allodynia in peripheral nerves. In our study, all three doses of the RAGE inhibitor blocked the diabetes-induced tactile allodynia, indicating that RAGE plays a role in at least some aspects of sensory nerve dysfunction in diabetes cases. Similar beneficial effects on tactile allodynia have been reported following inhibition of tumor necrosis factor in diabetic mice [52].

In conclusion, our data indicates that the novel fusion protein inhibitor of RAGE inhibited the diabetes-induced degeneration and permeability of the retinal vasculature and the dysfunction of peripheral sensory nerves. Interestingly, the inhibition of retinal capillary degeneration and permeability occurred at doses of the drug that did not correct several biochemical or physiologic abnormalities that have been postulated to contribute to the vascular disease. This difference in dose sensitivity between the effects on capillary leakage/degeneration and biochemical abnormalities should help focus attention on those abnormalities that are most strongly correlated with (and the likely cause of) the development of diabetic histopathology. RAGE continues to be a promising target for the pharmacologic treatment and prevention of diabetic complications in the eye and nerve.
